# Identifying social and economic barriers to regular care and treatment for Black men who have sex with men and women (BMSMW) and who are living with HIV: a qualitative study from the Bruthas cohort

**DOI:** 10.1186/s12913-017-2011-z

**Published:** 2017-01-28

**Authors:** Emily A. Arnold, John Weeks, Michael Benjamin, William R. Stewart, Lance M. Pollack, Susan M. Kegeles, Don Operario

**Affiliations:** 1 University of California San Francisco, 550 16th Street, San Francisco, CA 94114 USA; 2 CAL-PEP, 2811 Adeline St, Oakland, CA 94612 USA; 3 Brown University, 121 South Main St, Providence, RI 02906 USA

**Keywords:** HIV care continuum, Men who have sex with men and women, Structural factors

## Abstract

**Background:**

There is little research regarding the ability of Black men who have sex with men and women (BMSMW) to access and maintain HIV-related health care and treatment adherence. This population, who often insist on secrecy about their same-sex desire, may experience unique barriers to seeking regular care and treatment.

**Methods:**

From March 2011-April 2014, we recruited 396 BMSMW in the San Francisco Bay Area to be enrolled in our randomized controlled trial. At baseline we administered a behavioral survey assessing: demographics, homelessness, employment, history of incarceration, HIV status and disclosure practices, care and treatment adherence. 64 men reported living with HIV at intake. To learn more about their experiences, we recruited *N* = 25 to participate in qualitative interviews, which were conducted April-December 2014. Topics included: current living situation, diagnosis story, disclosure practices, experiences of accessing and maintaining care and treatment, and HIV-related stigma. Recordings were transcribed and coded for major themes.

**Results:**

Despite being located in an area where treatment is plentiful, men faced social and economic barriers to maintaining regular care and treatment adherence. Several findings emerged to shed light on this quandary: (1) Competing needs particularly around attaining stable housing, food security, and money created barriers to treatment and care; (2) Side effects of HIV medications discouraged men from adhering to treatment; (3) Provider and Institutional level characteristics influenced care engagement; (4) Disclosure and social support made a difference in care and treatment behaviors; and (5) Participants expressed a desire for group-based intervention activities to support treatment and care among HIV+ BMSMW. Inadequate engagement in the continuum of care for HIV was born out in the quantitative data where 28% of participants did not know their Viral Load.

**Conclusions:**

A holistic approach to HIV health for BMSMW would appear to translate to better outcomes for men living with HIV, where a goal of viral suppression must also include attending to their basic social and economic support needs.

## Background

Antiretroviral therapy (ART) has been shown to not only benefit those living with HIV but to dramatically reduce the likelihood of onward HIV transmission once individuals are virally suppressed [1]. Thus, “treatment as prevention” has been promoted as a core pillar of the National HIV/AIDS Strategy [2]. Yet, only a quarter of people living with HIV in the United States (US) have achieved the treatment goal of HIV viral suppression [3]. Indeed, there are significant gaps and health disparities along the HIV continuum of care, from HIV testing, to linkage and engagement in care, and to adhering to medication regimens. HIV continues to disproportionately impact Black Americans in the US, and Black men who have sex with men (BMSM) continue to experience the brunt of new cases annually and to experience higher rates of morbidity and mortality due to HIV/AIDS [4–6]. Recent studies have found that Black individuals enter care later in the course of the disease, are less likely to initiate care and ART, and are less likely to adhere to ART in comparison to White individuals [7, 8]. In the San Francisco Bay Area, Black men in both San Francisco and Alameda counties are linked at rates that are lower than men of other ethnicities, and they remain engaged in care, and achieve viral suppression, at lower rates when compared to White, Latino and Asian Pacific Islander men [9, 10]. For example, among new male HIV cases in San Francisco in 2013, 60% of Black men were virally suppressed, compared to 74% of Latino men, 69% of Asian Pacific Islander men, and 64% of White men. Black men experience disparities in HIV-related outcomes all along the care continuum [10]. Given the continued increase in cases of HIV infection among Black Americans, and BMSM in particular, it is urgent that prevention scientists develop interventions that increase regular engagement in care, access to ART, and use of support services that encourage adherence in this population [11].

Among BMSM, Black men who have sex with both men and women (BMSMW) have received little attention regarding their HIV care and treatment needs. BMSMW have complex sexual lives, with studies describing behavioral risk factors in this population that include multiple partners, inconsistent condom use, substance use in conjunction with unprotected anal and vaginal intercourse, and non-disclosure of their same-sex behaviors due to a need for secrecy and privacy [12–15]. Recent data indicate that BMSMW who are living with HIV are diagnosed later than other MSM, are more likely to be poor, and are more likely to experience barriers to accessing regular health care [16]. BMSMW are also less likely to be well informed about HIV, and are more likely to have concurrent sex partners [17].

A nascent literature on MSMW who are living with HIV has described their sexual risk behaviors, disclosure practices, substance use, and levels of depression as being different than those experienced by men who have sex with men only and men who have sex with women only [18]. MSMW living with HIV have been found to have higher Viral Loads than MSM, for example [19]. However, the literature examining engagement in HIV care and treatment among MSMW, and particularly among BMSMW, is sparse. BMSMW who do not identify as gay may not feel welcome in clinical settings catering to gay-identified men, and Black men in particular express distrust of the medical system [20], which has implications for tracking and encouraging positive clinical outcomes and medication adherence. In an era where “test-and-treat” represents the primary avenue towards controlling and reducing new HIV infections among BMSM especially, little is known about how BMSMW who are living with HIV navigate issues related to accessing and maintaining HIV treatment and care. Furthermore, it is not clear how BMSMW view viral suppression and their own ability to seek and maintain treatment over their lifetimes.

To develop an understanding of these issues, we embedded a qualitative study of BMSMW living with HIV into a larger HIV prevention trial called the “Bruthas Project”. This trial aimed to test a counseling intervention to reduce sexual risk behavior and increase regular HIV testing among those who were HIV-negative [21]. Notably, the Bruthas Project did not include any intervention activities to explicitly address the HIV-related health needs of HIV-positive men, and so the specific issues related to HIV-positive status were unaddressed in the design of the study. We sought to augment our understanding of the treatment and care experiences of HIV-positive BMSMW in our sample using qualitative in-depth interviews, with the overarching goal of informing subsequent intervention research focusing on the unique needs of this group.

## Methods

A full description of the Bruthas Project has been published previously [22]. Briefly, the intervention involved four individual counseling sessions with a Black male counselor in which participants discussed their sexual risk behaviors with female and male partners and examined the social and situational factors that influenced sexual decision making. Participants completed assessments at baseline and at 6- and 9-months post-enrollment to evaluate the intervention compared with a single-session control, and planned exit interviews following the 9-month assessment sought to examine participants’ experiences in the intervention program.

Of the *N* = 396 men originally recruited for a randomized controlled trial of Bruthas, 16% (*N* = 64) were living with HIV. From April-December 2015, we conducted in-depth interviews with *N* = 25 HIV-positive men who completed the Bruthas Project trial to examine their experiences as a man living with HIV and to elicit their ideas for a more tailored intervention to meet their unique needs. Participants were purposively selected based on their assignment to the intervention or control condition (with half of our sample recruited from each arm), self reported viral load (half self-reported being virally suppressed), and willingness to return for an additional interview. Baseline quantitative data from our participants were used to purposively select individuals to invite to participate in the qualitative interviews. Interviews were conducted by two Black male research associates (JW and WS), who were also the primary field staff for the Bruthas trial. Interviewers were trained in qualitative interviewing techniques and followed a semi-structured interview guide. Interviewers took field notes and wrote up interview summaries immediately following data collection, which the research team used to develop our analysis. The interview guide was initially piloted with three participants, then revised for specific wording and ordering of topics. Interview topics included: current living situation, diagnosis story, current access and use of HIV-related services and care, social support for living with HIV, their doctor-patient relationship, other health conditions, and suggestions for treatment and care interventions for HIV+ BMSMW. Participants received $35.00 at the end of their interview. Interviews were recorded and professionally transcribed. The Institutional Review Board at the University of California San Francisco reviewed and approved the study protocol. The study also complies with the COREQ guidelines and checklist for designing and disseminating the results of a qualitative study [23].

The research team (EA, JW, MB, WS) read through all interview transcripts and met weekly to discuss emergent themes and potential intervention implications based on the data, using principles from the Framework Analysis Method [24]. By including interviewers (JW and WS) as well as community-based service providers (MB) in this process, we were able to incorporate key field-based insights into our analytical framework. Following each meeting, the lead author (EA) wrote analytic memos summarizing the transcripts and group discussions to further develop theory and depth of understanding about the data. A codebook was developed that captured the primary themes and sub-themes, defining the themes within analytic codes, which were then applied to transcripts. The lead author (EA) and another research team member (JW) separately coded a total of 5 interviews to establish coder agreement, meeting weekly to discuss coding and resolve discrepancies in coding. Once 90% coder agreement threshold was achieved, the research team members (EA, WS and JW) coded the remaining transcripts using Dedoose, a cloud-based analytic program. Once the data were coded, the team searched Dedoose using particular codes, compiled relevant quotes and met again to discuss the results and to compare findings across the data set. The most relevant themes, in light of larger goals to achieve viral suppression among HIV-positive BMSMW, are presented below.

## Results

### Sample characteristics

The sample for the analyses presented here (*N* = 25) had a mean age of 50 (range 38 to 63), and the median time since diagnosis was 17 years 6 months (range 4 months to 27 years 9 months). Approximately half of the sample had received the Bruthas Project intervention (*N* = 12), and half had been enrolled in the control condition (*N* = 13). Quantitative baseline data indicated serious social and economic vulnerabilities within this sample: 17% were homeless in the past year, 84% experienced lifetime incarceration, and 83% were unemployed. In terms of lifetime incarceration and current levels of unemployment, the sub-sample reflected trends discerned in the larger cohort of Bruthas participants, however fewer reported being homeless in the past year than in the larger cohort (40%). In terms of clinical outcomes, 48% reported having a CD4 count between 500 and 1500, while 20% did not know their CD4 count. Almost half (48%) reported undetectable Viral Loads, while 28% did not know their Viral Load. These were comparable to reports in the larger sample of positive participants in the larger cohort.

Five key findings emerged from our qualitative data to shed light on the treatment and care experiences of our study sample: We discuss each key thematic finding in detail below.

### Competing needs and scarcity of affordable housing

Overall, we found that survival needs outweighed clinical care and adherence issues as the fundamental priority for participants. Many of our participants experienced substantial challenges to securing stable housing and sufficient food, and economic realities left quite a few needing to “hustle” or find work in the informal economy, through one time informal jobs or scams, in order to augment their income. In addition, even those receiving public financial support through Supplemental Security Income (SSI), Social Security Disability Insurance (SSDI), or General Assistance spent a great deal of time seeking out subsidized housing and food resources. Although participants were eligible for subsidized housing due to their HIV status, this resource was considered out of reach due to wait lists that could take months or even years to overcome.

Every place I applied for housing that is 30% of your Social Security they all have waiting lists. And I think they should have more buildings you know. Because rent is high. And some people can’t afford it you know. First and last deposit and they used to have places a long time ago that would help you but now they don’t. (Participant 004, 40–49 years old)

Multiple participants explained they missed medical appointments to participate in the informal economy or deal with unstable housing, and therefore they were out of care and not on treatment. Here one participant describes skipping care and treatment to attend to more immediate needs.

Hustle, trying to get a buck or two you know. If somebody have to go to the doctor’s appointment but you over here you want to make 20 dollars. Now if you don’t make that 20 dollars you don’t have nothing, you’ll go to the doctor and you’ll be hungry when you meet a doctor. And the doctor ain’t gonna give you 20 dollars but you know you’re gonna need it right now. So you gotta do something to put some money in your pocket. (Participant 015, 40–49 years old)

Another explains that his Social Security payments can barely cover rent in San Francisco and that he must supplement his income in order to “get through.” “*I’m on Social Security right now. $845 a month….[but] my rent is $725 every month…I recycle…And I cut grass, do yard work, you know that’s the only way I can get through*” (Participant 003, 60–63 years old). Generally, many of our participants did odd jobs, or collected cans to recycle, in order to make ends meet. Occasionally, a job would come up that would conflict with a doctor appointment, and participants prioritized dealing with their more immediate economic needs over their long-term health care.

Unstable housing and periods of living on the streets also contributed to episodes of missing treatment. For example, a homeless participant developed resistance to treatment after failing two regimens. He was diagnosed during a time when he lacked stable housing, and the chaos of his life made it difficult to remain adherent. Here he discusses losing his bag (with his medications inside) and also not wanting to take medication in front of others at the shelter because of HIV-related stigma.

Back then I was taking, oh goodness, it’s the blue pill. It was the two combination. Oh goodness, Sustiva -- and it was Sustiva and another one. It was a two pill combination. So when I came here they just kept me on that for a while. Then eventually I stopped taking it and I built a resistance. Then I was on Truvada, Reyataz and Norvir recently. And I was on it just too long…and I stopped because I wasn’t consistent. I was homeless. I had no place to stay. I didn’t know anybody you know…It’s important to have some type of stability if you’re going to take these medications…For the simple reason and fact that if you’re transient anything could happen. You can lose your bag, you can lose your medication. (Participant 013, 50–59 years old)

The material and economic circumstances, and especially the housing stability, of our participants impacted their ability to remain connected to care, regularly see their providers, and adhere to treatment regimens. SSI and SSDI payments, when received, were not enough to allow participants to pay rent in one of the most expensive urban centers in the US, and therefore they had to supplement their income with food from food banks and odd jobs, interfering with their ability to keep medical appointments. In extreme instances, participants lost their housing altogether and experienced quite concrete challenges to maintaining care and treatment if they were forced to live on the streets.

### Side effects and breaks in treatment

A number of participants had stopped taking their HIV treatment medications because of side effects or interactions with other medications. A few participants were not in care or on treatment because they “felt fine” and believed that medications would make them sick. Here one participant explains his distrust for pharmaceutical companies and resistance to treatment. Indeed, misinformation about medical regimens was part of the social context in which men were trying to adhere to treatment, making them distrust the impact of the drugs themselves. For example, this participant claimed the drugs made him sick:

I just didn’t think it was cool, man. I – it’s like, hell no. I mean it’s like, I’m healthy. I didn’t feel like I needed to [take treatment]. Then, come to find out I sent for this book ‘Everything You Thought You Knew About AIDS But Didn’t,’ man. I didn’t even know that the medication they was giving out was cancer treatment. The pharmaceutical companies, man were getting over like a fat cat in a pig farm. And so, you know what I’m saying, it was like, wow. Wow! So it was like – so I was really, really, hell, no I’m not taking no medication. And for a while, man my T cells, you know what I’m saying, just stayed the way that they were. They were cool…Viral Load didn’t even – the body didn’t even know what a Viral Load was. You know? It seems as if I did start taking this damned medication, man, that’s when all the stuff [changed]. Wow!

Q: You think there’s a connection when you started taking the meds this happened?

A: Yes, yes, yes, man! Yes! (Participant 012, 50–59 years old)

A number of older participants had a family history of diabetes and heart disease, and consequently were dealing with multiple chronic diseases alongside HIV. In several cases, this required men to take a variety of medications, some of which produced unsettling side effects including fatigue and depression, as described here:

A: Well, along with the HIV now I’m something like in the stage II diabetes thing to where it’s not out of control but they want to control it. But hey, man, I’m taking all these other medications for all this HIV and my heart disease and those side effects come in. So now I deal with them but it’s keeping my blood pressure down. I watch what I eat and everything else.

Q: What kind of side effects are you having?

A: Just like say sometimes I get fatigued real fast. Like during the day sometimes I might just be overwhelmed. And it’s even now when I stand up I get dizzy that I know that’s the diabetes thing. And then my vision is kind of going off now.

Q: And you also mentioned like that you’re reluctant to take some medications because you’re taking so many others already?

A: Yeah. I stopped taking – I cut everybody off for like 2 months, earlier this year. Yeah, doctors, and HIV meds. I got tired of taking them, man. And they just were, I’d just go pick them up and pop them in and I said, man. I just stopped taking them. I stopped taking everything…But you know I got into a depression state too. And every time I would open my cabinet I would see all these bottles. (Participant 018, 50–59 years old)

Here another participant talks about why he didn’t trust taking the medications prescribed to him by his doctor. And after he started his regimen, he believes adverse things started happening with his health.

Q: What kind of side effects would you get?

A: Nauseated, you know? Feeling sick, feeling lazy, don’t want to do nothing, you know? Feeling miserable. So I couldn’t take anything that would make me flip out. It started making me feel bad. I could feel bad already! You know? So why take a bunch of pills? I don’t even know nothing about the pills, really, to tell the truth. I was just doing what the doctors… what another man said.

Q: So they didn’t explain to you what the pills were?

A: Well they said, sometimes… you know, it’s kind of hard for me to believe the doctors sometimes. Because, in this day and age, and I’m not saying he’s a bad person, but see, when I went on dialysis, you know… I wasn’t having no pain or nothing. And I figured that he had me messed up for nothing, you know? He kept telling me to go to dialysis, calling my house, “You gotta go to dialysis. Your kidneys are bad.” Now the kidney is not giving me no problem so why should I go? And I’m thinking about all that stuff. I say, man, that messed my life up right there. I’m thinking about it. Because, a lot of times, I might not know as much as them. But a lot of times - I’ll be right. (Participant 015, 40–49 years old)

This participant clearly explains how ‘miserable’ he started feeling as a result of being on his medications. He also thought his HIV medications may have contributed to his kidneys failing and having to start on dialysis. Initially, he adhered to his regimen mainly because he was following his doctor’s orders. Eventually he stopped taking them because he believed they were worsening his health.

This participant lives on SSI payments ($845/month), which he qualified for based on his HIV-related disability. At the time of the interview, he was insured through Medicaid (a publicly-funded form of health insurance) and had recently changed doctors. Below, he describes the impact of taking 27 pills a day (the regimen that his previous doctor had prescribed for him) on his physical and mental health. He complained that taking that many pills had a significant negative impact on his ability to function throughout the day, including his ability to work.

A: I couldn’t function [taking so many pills]. I couldn’t – I was always asleep. I couldn’t function or do nothing.

Q: Did you ever talk about these issues with your doctor who was giving you the 27 pills?

A: Yes.

Q: And what was his response to that?

A: He said in time it would get better. And that was it. And so, because even people who wasn’t a doctor, they was telling me, “You’re taking too many pills!” (Laughs) Even my therapist told me, “You’re taking too many pills. And you need to change doctors.” So I said, “Okay.” (Participant 004, 40–49 years old)

This participant faithfully adhered to the regimen prescribed by his doctor even though taking 27 pills a day had a significantly negative impact on his health and general ability to function. Eventually, he was empowered by those around him to change doctors. Once he did that, he claimed that his regimen was immediately reduced.

### Relation to care: provider-level and institutional characteristics

We found that there were characteristics of providers and medical institutions that were associated with whether or not BMSMW were engaged in care and on treatment.

### Provider characteristics and relation to care

Participants who were retained in care and adherent to their treatment regimens attributed their health behaviors to a strong relationship with their doctor, which was characterized by open communication and trust.

[My doctor] is a foreign woman. I don’t know where she’s from, but… she’s really nice and personal. Because I had kinda, like, been slipping on my pills. Cuz I had been undetectable for a while, you know. And then I went there and then I wasn’t undetectable anymore. And she was like, “You’re not taking your pills! I know that’s what it…” And she got on me, you know! She really cared, you know? And I really I loved that…And it made me start taking my pills. Because, I was saying, you know, I don’t want to go back and she’d be like, “You promised me all this," and I didn’t want to disappoint her over myself. (Participant 020, 50–59 years old)

The same participant had switched from a large public hospital to this smaller clinic because he felt that the staff “really cared” about him and would call him if he missed appointments repeatedly.

If they ain’t seen me in a while they’ll call me and you know see what’s going on, you know, pretty much just show someone that they care, you know, that they really care about whether they’re doing their job, you know. That’s why I left [a large public hospital] because [they] never checked on me. And if they hadn’t seen me in a while or call me and say it’s time for your flu shot or it’s time for your, you know your Hep shot and all this. But you know [the new clinic staff] care, that’s why I go there cuz they care. And they make me feel like they care so that’s all that matters. That’s all that matters to me. (Participant 020, 50–59 years old)

Another participant receives care at a large university hospital and credits the relationship he has with his doctor, who is an African American man, with enabling him to stay in care and on treatment. Here he describes their rapport, based on shared cultural understandings and traditions.

[The clinic is for] all Black men. And they treat us accordingly, I guess you know. I guess we’re different from everybody [else]…I ain’t never had a doctor like him. Never…Yeah, well… now I’m not prejudiced or nothing, but I feel like if you never walked in a Black man’s shoes, you don’t know what we go through. So, he’s an African-American doctor, so I can’t fool him. He’ll be like, “Man, please!” Do you know what I mean? Yeah, I can’t tell him nothing. But if I have a White doctor or a Mexican doctor, something like that? They care but they *don’t care*, do you know what I mean? You’re just a patient there… you’re a patient. My doctor? We’re personal. We’re real like that, yeah. (Participant 021, 30–39 years old)

This participant underscores the importance of having medical providers who reflect the ethnic and racial composition of the patients that they care for.

### Institutional characteristics and relations to care

In contrast to the participant above, another participant was not on treatment at the time of the interview. He was having a challenging time identifying a doctor he could trust; because of this distrust, he felt unsure about initiating treatment. His providers were part of a large Health Maintenance Organization (HMO), which he felt was “indifferent” to him. Here he describes his search for an appropriate doctor:

Q: How do you like [Large HMO]?

A: I’m indifferent to [Large HMO]. They’re indifferent to me and I’m indifferent to them. It’s a struggle, you know? To want to do things my way and they want to do it their way. And there’s no meeting in the middle, unless there is a crisis.

Q: What is their way? When you say ‘their way’?

A: Well, they have rules and regulations. They don’t go outside the box. It’s [their] way or no way, you know? But, you know, I don’t necessarily want to go that route. I want more community involvement, more groups, you know? For things not to be so rigid! With [large HMO], everything’s very rigid. And I’ve struggled… I’m struggling to find that right doctor. And they think I’m doctor shopping. But I’m not! I’m just trying to find the right doctor. I’m not taking any medications. So, therefore, how can I be doctor shopping? So, I don’t want to start taking medications with someone I don’t trust, you know? (Participant 019, 40–49 years old)

Some individuals also experienced differences in care with expansion of Medicaid and a shrinking network of providers who were willing to see them, resulting in long wait times at clinics and very brief and impersonal visits with their doctors. Here a participant on Medicaid found that the wait times at his provider’s clinic were as long as 2–3 h. Here he describes cancelling appointments due to the wait time:

The only problem I have with it is waiting…But, I think a lot of these clinics, when you don’t have - -when you didn’t have, like, [Medicaid] like I have now, you had to wait. Because if you were getting, like, [the county-run health insurance plan] or whatever, there were only certain places you could go and use that. So, I know I have to be there 2 or 3 h sometimes. And I hate going. So, sometimes I don’t feel like it and I cancel the appointment. Like I did the last two appointments I’ve had. (Participant 007, 40–49 years old)

Finally, one more participant stayed with the same doctor and clinic for 10 years while his care team searched for a way to help him control his HIV. This participant has been living with HIV for over 20 years and is connected to a number of services, including housing and a peer-led group for HIV-positive Black men. Here he explains that he stayed connected to this particular clinic because the providers facilitated his access to clinical research studies, which enabled him to be part of trials that tested different regimens. Eventually, he was placed on his tenth treatment regimen, which finally worked.

Yeah. And at first they… well, my T-cells? It was like 100. Or 150. We done tried everything… I’ve been in every medical study, you know, to try to find out. And the last one I was in, we found the regimen that works for me. And that’s what they tell me, you know, I usually tell everybody, “I’ll be the guinea pig. I’ll be the guinea pig.” And now you know they found a regimen that works for me so my T-cells went up, my Viral Load is undetected. And I haven’t had no real health problems in the last, what, 10 years? (Participant 009, 50–59 years old)

Having a good relationship with one’s doctor and feeling like the clinic could be trusted was a key in allowing men to stay connected to care and in treatment.

### Disclosure and social support for living with HIV

Although some participants had disclosed their HIV status to friends, family, or sexual partners, roughly a third were more secretive about their status and therefore were receiving little to no social support for remaining in care. Although a few men had widely disclosed and therefore had extraordinary levels of social support from friends, family and formal support groups for living well with HIV, others were coping with their HIV alone and in relative isolation. These men felt it was hard to disclose to family and friends, due to anticipated HIV-related stigma and fear of rejection. Also some did not disclose until they were catastrophically ill and in the hospital. However, for the few who were able to disclose and elicit support from family or friends, the help they were able to access impacted their well-being and ability to stay on treatment and in care.

Q: How much support do you have now?

A: Um, my family and my extended family, nieces and nephews, great niece and nephew, you know, they all know. And they’re very supportive, you know. I talk to them, you know, maybe twice, maybe three times a week and they always ask me, you know, “How you doing with your HIV? Are you taking your meds?” You know. “Are you doing okay?” That’s just the way they are now. (Laughs) So, that makes me feel… I mean, I feel, you know, that made me feel good. I felt good about that. In comparison to, you know, almost 20 years ago, it’s like… (Chuckles)

Q: And as far as outside the family, what other kind of support do you have?

A: I attend a support group… people dealing with HIV. It just keeps me – it keeps me grounded. It makes me… even though I’m doing really well, but you know, I still have to acknowledge the fact that I’m still dealing with this. And I need to be aware of that. It just makes me – it makes me stronger. (Participant 016, 50–59 years old)

Another participant had a female friend who was also HIV-positive and helps him navigate services, including successfully applying for SSDI. This participant also had excellent family support:

Then I was just in a drug whirlwind for like –For like 4 years after that. And then…my friend came into my life and, you know, I got sick…I knew her for a while. I didn’t know she was HIV though. She was HIV before me. And then when my parents passed, she was there for me. And then that’s when …I found out she was also HIV. And so I was able to talk. I had somebody to talk to. That was a blessing, man! Thank God for her, boy! I can tell her about my gay side of life and she was just heaven sent to me, man. But… and then, you know, I kinda got sick and in, like, 2005 or’06 or something…She’s the one helped me get on [Social Security Disability Insurance] too! Yeah, and then of course the first time they turn you down, and I was ready to give up. And she told me, “No!” – I remember that – “No! Don’t give up.” You know? “They’re going to make you fight for it.” And I got it, you know….Oh, it’s lovely, man!…I don’t have no problem with food, you know? Because… I go to my brother’s house and cook. Because I ain’t got no kitchen. I ain’t got no stove or nothing. And all I got is a microwave, man! So, I go to my brother’s every weekend and cook. And then my grandma, you know a grandmother… She’s always hooking me up with stuff….So, I got a good home at home, you know? A foundation. And my whole family knows about me. And they support me and, you know… it’s good. (Participant 020, 50–59 years old)

In contrast to the participant above, another participant was extremely isolated and unable to tell anyone about living with HIV. He feared disclosing “his business” and having his HIV status become known by associates also living in the neighborhood where he resided. After deciding that he could no longer “sneak” to take his medications, and “giving up," he became so ill that he was checked into hospice:

Well, you see, I wasn’t taking my medicine. And stuff like that and my T-cells were gone. And I mean, I was just giving up, you know? So, then I went there [to hospice] and came back….I just got tired of, you know, every time I’m with somebody… man, I gotta take my medication. I gotta tell them what I gotta do. And then I gotta sneak and take it, you know? I just don’t want to tell nobody my business right now. (Participant 015, 40–49 years old)

This individual explains that he did not want others knowing about his health situation, and so he stopped taking his medications. Eventually his health deteriorated to the point where he had no T-cells left and he was admitted to a hospice program where he reinitiated treatment.

These social dynamics demonstrate the impact that disclosure and social support for living with HIV can have for participants. In cases where men were able to disclose their HIV status, especially to others who were also living with HIV, they were able to get support for staying on treatment, for being in care, and for being connected to social resources designed to help those with HIV. Participants without such strong support did not do as well, and some came close to dying due to adherence-related issues.

### Group-level and peer support activities

The majority of our participants expressed a desire to join a support group made up of other BMSMW so they could discuss matters related to living with HIV, being BMSMW, and remaining engaged in care and treatment. This finding emerged in response to questions about the best format for an intervention, and was surprising due to the emphasis on the need for privacy and confidentiality for BMSMW in our earlier intervention development work. As described, the Bruthas Project was designed as an individual level, counseling-based behavioral risk reduction intervention, but men who were living with HIV urged the study team to consider adding an additional small group component for the purposes of providing informational and emotional social support to BMSMW living with HIV. For example, one participant was diagnosed late and his doctor informed him during the initial diagnosis that if he had waited another month he would have developed full blown AIDS and likely died. Consequently, this participant was depressed and felt that treatment was futile. Eventually, he joined a support group with other Black men, where he felt “hope.”

Because at one point, I said, “Hey, I don’t even want [treatment]… forget it, man! It’s done! It’s over!” You know? But now, I finally just went on and started taking the cocktail. I started feeling good about myself. And just listening to other people’s stories about how they dealt with it and how they went through that transition. And then seeing how healthy they was and stuff like that, that gave me a sense of hope. (Participant 005, 40–49 years old)

Several men noted that it would be helpful to talk to a group of people who “had the same problems” and who were also struggling to take treatment regularly and continue seeing their medical providers. This participant notes that the space used for a support group would need to make everyone feel welcome, not be conspicuous as a place people living with HIV go to, and convenient.

You know there should be some programs, some good programs out there for people that’s really embarrassed and actually don’t want to be seen in them [HIV-identified] places. That’s one reason why I left [Hospital X] because there’s too many people coming in there. People be looking at you, talking about folks and Lord, you know I don’t care but I don’t want my business out on the street. (Participant 020, 50–59 years old)

Participants expressed a desire for group sessions with other BMSMW that centered on information sharing, but that also had a component related to social support and empathy for “having the same problems.” Importantly, Participant 020 clearly states that he does not want to “be seen in them places” which are more HIV-specific. Furthermore, he does not want others sharing “his business” with others in the community. The need to maintain confidentiality about one’s HIV status was important for many in our cohort, who were reluctant to be seen in HIV-identified spaces.

## Discussion

In this qualitative examination of the treatment- and care-related needs of BMSMW living with HIV, we found that participants are prioritizing their basic, more immediate needs, such as food security and housing, ahead of maintaining their provider appointments and medications. Indeed, we observed problems accessing and limitations in Social Security and other benefits for this socioeconomically disadvantaged sample, which undermine their ability to focus on their health. In the San Francisco Bay Area, as in other global urban centers, housing costs have skyrocketed in the past several years and affordable housing is increasingly scarce [25], even for the middle class, which poses structural challenges in an environment known for having progressive policies toward HIV care and treatment. Thus, a purely biomedicalized approach to disrupting the HIV epidemic, such as treatment as prevention, may find limited success in the absence of programmatic attention to the social conditions that enable healthcare utilization and facilitate medication adherence. Taking a tailored, individualized approach towards helping men themselves identify and successfully garner resources that will allow them to meet their basic survival needs is one strategy to empower and enable a socioeconomically disadvantaged population, such as the sample for this study, to remain in care and on treatment.

In addition to economic factors, HIV service and medical professionals must also be aware of co-occurring health conditions such as substance use and mental health issues which can create challenges in attaining stability as well as continuing on treatment. Funding policies and programs, such as those offered through the Ryan White Care Act, are essential to provide socioeconomically disadvantaged people living with HIV, such as those in our sample, access to wrap-around services, including social workers and case managers, as well as integrated models of care, where mental health and substance use treatment can be accessed in the same location as HIV specialty care [26, 27].

Our participants also experienced a number of severe health conditions owing to interactions between their chronic disease medications and their ART. We found this especially problematic among older segments of the study sample who are managing aging and chronic disease along with HIV-specific concerns such as treatment regimens and side effects. Whereas other investigators have examined co-morbidities of HIV and aging among African American women [28], little research has been conducted with African American men. Among women, HIV and co-morbidity self-management have been found to be linked, and those who are more socially isolated reported less optimal adherence and management [29]. Other studies have reported that patients with co-morbidities delay ART initiation [30]. Participants in this sample reflected that, as our population living with HIV is aging, group-based programs may help to educate Black MSMW living with HIV about various types of HIV treatments and side effects, how these might interact with other medications for chronic diseases, and create opportunities for support. Groups could potentially provide space for individuals to rehearse provider communication in order to empower them to request changes in their medications when needed. Such HIV support groups for BMSMW could also be held in settings away from hospitals and locales that people with HIV access, in order to avoid inadvertent disclosure of participant HIV statuses.

Many of our participants who were in treatment and regularly seeing a provider reported having a positive relationship with their doctor and social support for living with HIV. Positive provider relationships, which include the ability to be open and trusting with one’s provider, and peer support have been associated with better treatment adherence in qualitative studies with African American women [31]. However, African American men in particular have experienced negative encounters with medical professionals [20]. For our participants, as in other studies, having providers who were culturally competent with BMSMW, and who often were African Americans themselves, allowed a deeper level of trust and honesty to develop. Improving cultural competency within the provider workforce, and increasing the diversity of the workforce and the number of medical providers who are African American themselves, may help BMSMW living with HIV to overcome barriers to remaining in care and on treatment as they feel more comfortable forging relationships with their providers. More research on targeting programs that increase diversity among healthcare professionals may lend crucial insights into meeting this unmet need for BMSMW.

Finally, we also observed the important role of social support for those living with HIV. Participants who were able to share their health status with family or with friends expressed a more positive outlook on their capacity to navigate HIV treatment and care and social service systems. With disclosure comes the ability to access social support, which came in the form of food assistance, assistance with keeping medical appointments, and assistance navigating social services designed to support those with HIV. Investigators have observed similar patterns among heterosexually-identified African American men living with HIV, in which social support and positive coping were associated with better physical and global functioning [32]. All of the men in this sample endorsed the idea of support groups for BMSMW living with HIV, which may offer new directions for future interventions.

### Limitations

This was a purposive sample of men living with HIV who were enrolled in a randomized controlled trial of an intervention designed for BMSMW. Participants interviewed in this study had completed at least one session of counseling for HIV risk reduction, and thus may have had greater comfort discussing their experiences living with HIV compared with peers without this opportunity. It is also likely that the perspectives of men enrolled in an HIV prevention intervention trial are different than BMSMW in the general population. In addition, the study was conducted in a geographic region known for having assertive health policies related to HIV care and treatment, as well as toward sexual and gender minority populations. However, it is likely that if our participants were experiencing barriers to accessing care and treatment in San Francisco, one of the best possible social and healthcare settings for BMSMW living with HIV, they will experience barriers elsewhere. We believe that the findings may suggest important barriers and facilitators to accessing care and remaining on treatment for BMSMW who are living with HIV, however due to the relatively small size of this qualitative study, the findings cannot be generalized to the larger population of BMSMW.

## Conclusions

No interventions for addressing the treatment- and care-related needs or other health co-morbidities exist for HIV-positive BMSMW. The Bruthas Project is an individual-level intervention focusing on behavioral risk reduction for preventing HIV transmission, and did not contain activities that were directly designed to support men living with HIV. Although initial findings from the Bruthas trial suggest some promise, HIV-positive MSMW who participated in the trial indicated that they experienced structural barriers to remaining in care and on treatment due to poverty and unstable housing, and several reported discontinuing their HIV treatment medications due to interactions with medications for other co-morbidities. These emergent findings suggest the need to design tailored intervention strategies for HIV-positive BMSMW, which may include group-level activities to increase social support for them, and to improve their treatment literacy. Intervention development for this population must address the socioeconomic context that might challenge engagement in clinical care and medical adherence, support BMSMW to establish and maintain positive relationships with medical providers, and provide social support for living with HIV.

## Abbreviations

AIDS: Acquired immunodeficiency disease syndrome
ART: Antiretroviral therapy
BMSM: Black men who have sex with men
BMSMW: Black men who have sex with men and women
HIV: Human immunodeficiency virus
HMO: Health maintenance organization
SSDI: Social security disability insurance
SSI: Supplemental Security Income
US: United States

## References

[1] Cohen MS, McCauley M, Gamble TR (2012). HIV treatment as prevention and HPTN 052. Curr Opin HIV AIDS.

[2] The White House Office of National AIDS Policy. National HIV/AIDS Strategy for the United States. Washington, DC: The White House Office of National AIDS Policy; 2010.

[3] Morin SF, Kelly JA, Charlebois ED, Remien RH, Rotheram-Borus MJ, Cleary PD (2011). Responding to the National HIV/AIDS Strategy-setting the research agenda. J Acquir Immune Defic Syndr.

[4] Centers for Disease Control and Prevention. HIV Surveillance Report. Atlanta, GA: Division of HIV/AIDS Prevention, National Center for HIV/AIDS, Viral Hepatitis, STD, and TB Prevention; 2012.

[5] Millett GA, Peterson JL, Flores SA, Hart TA, Jeffries WL, Wilson PA, Rourke SB, Heilig CM, Elford J, Fenton KA (2012). Comparisons of disparities and risks of HIV infection in black and other men who have sex with men in Canada, UK, and USA: a meta-analysis. Lancet.

[6] Prejean J, Song R, Hernandez A, Ziebell R, Green T, Walker F, Lin LS, An Q, Mermin J, Lansky A (2011). Estimated HIV incidence in the United States, 2006–2009. PLoS One.

[7] Hightow-Weidman LB, Jones K, Phillips G, Wohl A, Giordano TP (2011). Baseline clinical characteristics, antiretroviral therapy use, and viral load suppression among HIV-positive young men of color who have sex with men. AIDS Patient Care STDs.

[8] Thames AD, Moizel J, Panos SE, Patel SM, Byrd DA, Myers HF, Wyatt GE, Hinkin CH (2012). Differential predictors of medication adherence in HIV: findings from a sample of African American and Caucasian HIV-positive drug-using adults. AIDS Patient Care STDs.

[9] Alameda County Department of Health (2016). HIV in Alameda County 2012–2014.

[10] San Francisco County Department of Health (2015). HIV Epidemiology Annual Report 2014.

[11] Maulsby C, Millett G, Lindsey K, Kelley R, Johnson K, Montoya D, Holtgrave D (2013). A systematic review of HIV interventions for black men who have sex with men (MSM). BMC Public Health.

[12] Lapinski MK, Braz ME, Maloney EK (2010). The down low, social stigma, and risky sexual behaviors: insights from African-American men who have sex with men. J Homosex.

[13] Harawa NT, Williams JK, Ramamurthi HC, Manago C, Avina S, Jones M (2008). Sexual behavior, sexual identity, and substance abuse among low-income bisexual and non-gay-identifying African American men who have sex with men. Arch Sex Behav.

[14] Bingham TA, Harawa NT, Williams JK (2013). Gender role conflict among African American men who have sex with men and women: associations with mental health and sexual risk and disclosure behaviors. Am J Public Health.

[15] Operario D, Smith CD, Arnold E, Kegeles S (2011). Sexual risk and substance use behaviors among African American men who have sex with men and women. AIDS Behav.

[16] Singh S, Hu X, Wheeler W, Hall HI (2014). HIV diagnoses among men who have sex with men and women-United States and 6 dependent areas, 2008–2011. Am J Public Health.

[17] Dyer TP, Regan R, Pacek LR, Acheampong A, Khan MR (2015). Psychosocial vulnerability and HIV-related sexual risk among men who have sex with men and women in the United States. Arch Sex Behav.

[18] Jeffries WL (2014). Beyond the bisexual bridge: sexual health among U.S. men who have sex with men and women. Am J Prev Med.

[19] Friedman MR, Stall R, Silvestre AJ, Mustanski B, Shoptaw S, Surkan PJ, Rinaldo CR, Plankey MW (2014). Stuck in the middle: longitudinal HIV-related health disparities among men who have sex with men and women. J Acquir Immune Defic Syndr.

[20] Malebranche DJ, Peterson JL, Fullilove RE, Stackhouse RW (2004). Race and sexual identity: perceptions about medical culture and healthcare among Black men who have sex with men. J Natl Med Assoc.

[21] Operario D, Smith CD, Arnold E, Kegeles S (2010). The Bruthas Project: evaluation of a community-based HIV prevention intervention for African American men who have sex with men and women. AIDS Educ Prev.

[22] Arnold EA, Operario D, Cornwell S, Benjamin M, Smith CD, Lockett G, Kegeles SM (2015). The Development of a Counseling-Based HIV Prevention Intervention for African American Men Who Have Sex With Men and Women: The Bruthas Project. AIDS Educ Prev.

[23] Tong A, Sainsbury P, Craig J (2007). Consolidated criteria for reporting qualitative research (COREQ): a 32-item checklist for interviews and focus groups. Int J Qual Health Care.

[24] Ritchie J, Lewis J (2003). Qualitative Research Practice: A Guide for Social Science Students and Researchers.

[25] Whittle HJ, Palar K, Hufstedler LL, Seligman HK, Frongillo EA, Weiser SD (2015). Food insecurity, chronic illness, and gentrification in the San Francisco Bay Area: An example of structural violence in United States public policy. Soc Sci Med.

[26] Beane SN, Culyba RJ, DeMayo M, Armstrong W (2014). Exploring the medical home in Ryan White HIV care settings: a pilot study. J Assoc Nurses AIDS Care.

[27] Cahill SR, Mayer KH, Boswell SL (2015). The Ryan White HIV/AIDS Program in the Age of Health Care Reform. Am J Public Health.

[28] Prado G, Feaster DJ, Schwartz SJ, Pratt IA, Smith L, Szapocznik J (2004). Religious involvement, coping, social support, and psychological distress in HIV-seropositive African American mothers. AIDS Behav.

[29] Warren-Jeanpiere L, Dillaway H, Hamilton P, Young M, Goparaju L (2014). Taking it one day at a time: African American women aging with HIV and co-morbidities. AIDS Patient Care STDs.

[30] Abara WE, Smith L, Zhang S, Fairchild AJ, Heiman HJ, Rust G (2014). The influence of race and comorbidity on the timely initiation of antiretroviral therapy among older persons living with HIV/AIDS. Am J Public Health.

[31] Okoro O, Odedina FT (2016). Improving medication adherence in African-American women living with HIV/AIDS: Leveraging the provider role and peer involvement. AIDS Care.

[32] Shah K, McMahon JM, Trabold N, Aidala AA, Chen M, Pouget ER, Simmons J, Klostermann K (2015). Determinants of physical and global functioning in adult HIV-positive heterosexual men. AIDS Care.

